# Uplifts and hassles are related to worsening in chronic fatigue syndrome: a prospective study

**DOI:** 10.1186/s12967-023-04412-z

**Published:** 2023-08-20

**Authors:** Fred Friedberg, Jenna L. Adamowicz, Patricia Bruckenthal, Maria Milazzo, Sameera Ramjan, Xiaoyue Zhang, Jie Yang

**Affiliations:** 1 Renaissance School of Medicine, Stony Brook University, Stony Brook, New York United States; 2 Psychological and Brain Sciences, University of Iowa, Iowa City, Iowa United States; 3 School of Nursing, Stony Brook University, Stony Brook, New York United States; 4 Memorial Sloan Kettering Cancer Center, New York, New York United States

**Keywords:** Uplifts, Hassles, Social events, Worsening, Chronic fatigue syndrome

## Abstract

**Background:**

Limited published data suggests that absence of uplifts (minor pleasant events) is associated with clinical worsening in patients with chronic fatigue syndrome (CFS). The current study aimed to assess the relation of illness worsening to the trajectories of social and non-social uplifts and hassles in a six-month prospective study in CFS.

**Methods:**

Participants were primarily in their 40s, female, white, and ill for over a decade. All participants (N = 128) met criteria for CFS. The interview-based global impression of change rating was used to classify individual outcomes as improved, unchanged, or worsened at six- month follow-up. Uplifts and hassles, both social and non-social, were assessed with the Combined Hassles and Uplifts Scale (CHUS). The CHUS was administered weekly in online diaries over six months. Linear mixed effect models were utilized to examine linear trends for hassles and uplifts.

**Results:**

No significant differences were found between the three global outcome groups for age, sex, or illness duration; however, work status was significantly lower for the non-improved groups (*p* < 0.001). Non-social hassles intensity showed an increasing slope for the worsened group (*p* = 0.03) and a decreasing slope (*p* = 0.05) for the improved group. For the worsened group, a downward trend was found for frequency of non-social (*p* = 0.01) uplifts.

**Conclusion:**

Individuals with worsening as compared to improving illness in CFS show significantly different six-month trajectories for weekly hassles and a deficit in uplifts. This may have clinical implications for behavioral intervention.

*Trial registration* ClinicalTrials.gov ID: NCT02948556.

## Background

Psychological uplifts are minor pleasant events, such as completing a rewarding task, that occur in daily life [1]. Although these events appear to be important to well-being [2], they have not been extensively studied. By comparison, minor stressors or “hassles”, such as misplacing things, have received considerably more empirical attention. Hassles have been associated with increased somatic health symptoms, e.g., backaches, headaches [1], as well as decreases in health and positive mood, whereas uplifts can make a person feel joyful, glad, or satisfied [2]. Uplifts and positive events are correlated with lower fatigue in individuals with chronic fatigue and chronic pain [3, 4].

Uplifts and hassles also appear to have biobehavioral effects. A cross-sectional study of healthy adults [5] suggested that hassles and uplifts significantly and independently predicted changes in inflammation markers [e.g., Interleukin-6 (IL-6)], independent of sociodemographic, biological, and psychological measures, including depressed mood. A later prospective study of over 900 middle-aged adults [6] found that the frequency of daily positive events was associated with lower inflammatory markers (IL-6 and C-Reactive Protein) in the overall sample, and lower fibrinogen among women. Effects were more pronounced for participants in the lowest quartile of positive event frequency, suggesting that lack of positivity in daily life may be particularly consequential for inflammation. Furthermore, interpersonal positive events were more predictive of lower IL-6 overall and lower fibrinogen in women than non-interpersonal positive events. The authors concluded that daily positive events may serve a protective role against inflammation, a biological factor which may contribute to the pathophysiology of particular subgroups in chronic fatigue syndrome (CFS) [7].

Apart from biological correlates, several behavioral papers [8] suggest that social interactions may play a role in determining the magnitude of fatigue experienced by those with chronic pain [9]. Specifically, investigations of rheumatoid arthritis, osteoarthritis, and fibromyalgia (FM) patients have shown that positive interpersonal events are associated with lower daily fatigue and negative interpersonal events are correlated with elevated daily fatigue [9, 10]. Furthermore, the impact of hassles may also play a role in negative outcomes. A cross-sectional study [11] comparing newly diagnosed CFS and FM patients to multiple sclerosis and arthritis patients found that the combined CFS and FM group showed a higher frequency and higher emotional impact of daily hassles. This may indicate a need for better coping with hassles and/or positive behavioral changes that may reduce hassles as part of a self-management program [12]. These reported associations between commonly experienced positive and negative events, and fatigue symptoms in chronic pain and chronic fatigue conditions suggest that clinical approaches to potential illness improvement may be enhanced with careful assessments of these interactive phenomena.

Recently, a six-month observational study of a biobehavioral model in CFS [13] found that decreased intensity of behavioral uplifts, as assessed on the Combined Hassles and Uplifts scale (CHUS) [14], was the only significant behavioral predictor of patient-reported global non-improvement. Given this intriguing, if somewhat imprecise finding, perhaps the CHUS measure could be more informative if greater specificity could be applied to its constructs. For instance, in a study on relationship satisfaction [15], hassles and uplifts on an abbreviated version of the CHUS were grouped into those dealing with social (e.g., family, friends) and non-social (e.g., job, health) events. Contrary to their hypothesis, *non-*social uplifts had the strongest positive impact on relationship satisfaction. In CFS, the influence of these minor social and non-social events may shed light on their relative importance in influencing outcomes.

With respect to longer-term outcomes, positive impacts of uplifts have been reported in a one-year prospective study of 130 patients with chronic fatigue syndrome (CFS) [3]. This study found that pleasant activities and/or life events implying moderate or major life changes were associated with significantly improved outcomes, including reduced fatigue and impairment. Similarly, a clinical model of behavioral intervention in CFS [12] suggested therapeutic prescription of uplifting activities and the enhancement of positive coping skills to diminish the impact of hassles and improve outcomes. These clinical research threads may have implications for better-targeted approaches to behavioral management for patients with fatiguing illness.

The purpose of the current report was to assess the relation of the global outcomes of illness worsening and improvement to the trajectories of social and non-social uplifts and hassles in a six-month prospective study in CFS. Although the global outcome rating is frequently used as an important indicator of perceived change in CFS observational and intervention studies [16–18], its relation to potentially influential patterns of uplifts, hassles, and social and non-social events has not been studied. Furthermore, validated weekly assessments, rarely reported in CFS observational studies, may have utility in identifying specific behavioral patterns that may influence outcomes, particularly illness worsening that, in turn, may inform therapeutic management strategies.

## Methods

### Participants and procedure

This report utilized data from a six-month home-based observational study in 128 CFS patients, detailed elsewhere [13] that examined biobehavioral predictors of global outcomes. Most participants were in their 40s (*M* age = 46.11, *SD* = 11.8), female (87.2%), white (90.3%), unemployed or on disability (67.9%), and ill with CFS for over a decade (*M* = 16.5 years, *SD* = 10.3). Baseline questionnaire scores showed clinically relevant fatigue severity (Fatigue Severity Scale; [19]), impaired physical function (SF-36 Physical Function Subscale; [20]), and elevated autonomic symptoms (COMPASS; [21]. The entire study sample met symptom and impairment criteria for CFS [22], as assessed in a validated phone interview [23] conducted by research nurses (PB, MM) experienced in chronic fatigue and chronic pain assessments.

The primary study protocol [13] (Table 1) classified subjects into improved and non-improved groups with behavioral predictors (e.g., uplifts) based on 26-week means. The current study divided the CFS sample into three outcome groups, i.e., improved, unchanged, and worsened and created new variables for social and non-social uplifts and hassles. Weekly uplifts and hassles scores drawn from assigned web diaries were utilized in the data analysis as behavioral predictors of outcomes across the three outcome groups, which were treated as response variables in our models.

**Table 1:** Protocol of primary study

• Baseline questionnaires
• 26 weekly web diaries for symptom and stress ratings, activity patterns, hassles and uplifts
• 26 weekly collections of heart monitor data (autonomic activity)
• Initial 13 weeks of waking actigraphy data collection
• Six-month follow-up interviews for functional assessment and global change ratings
• Data analysis of biobehavioral predictors of subjects classified into “improved” and “non-improved” groups

Nationwide recruitment in the United States began in September, 2016 and ended in October, 2019. Recruitment methods included study announcements posted on major CFS patient support websites (e.g., Health Rising, SolveME) and in the large private practices of CFS specialized physicians located in New York and Utah. Without a travel requirement, this home-based study was considered more likely to recruit these under-served patients, particularly those who were disabled and homebound [24]. This study was approved by the Stony Brook University Committee on Research Involving Human Subjects which reviewed and approved the study procedure. All participants provided written informed consent via land mail of signed consent forms. Participants were compensated up to $300 for their participation. The study was pre-registered on ClinicalTrials.gov (NCT02948556).

### Measures

#### Hassles and uplifts

Hassles and uplifts were measured with the Combined Hassles and Uplifts Scale (CHUS) [14]. A 26-week weekly web diary (ScienceTrax, Inc., Macon, Georgia) contained the 53-item CHUS which measures perceived hassles and uplifts. Hassles are defined as “irritants—things that annoy or bother you; that can make you upset or angry”. Uplifts are defined as “events that make you feel good; that can make you joyful, glad, or satisfied”. The CHUS yields subscales of frequency and intensity. Hassles and uplifts frequency scores have a potential range of 0 to 53, with the total score indicating how many items were simply endorsed. When endorsing an item, participants are asked to rate how much of a hassle or uplift the specific item was. Items are rated on a 3-point Likert-based scale ranging from 1 (*somewhat*), 2 (*quite a bit*), to 3 (*a great deal*). The average rating of these items yields intensity scores. Participants may rate events as hassles, uplifts, or both. The CHUS has shown good reliability and validity in predicting mood and somatic health outcomes [11, 25]. The measure has high test–retest reliability and a reported Cronbach's alpha of 0.71 [2]. The CHUS alpha for the present study was excellent (α = 0.87).

Based on a prior study of relationship satisfaction [15], the CHUS items were subdivided into ten social (e.g., children, relatives, family obligations, friends) events and 43 non-social (e.g., job, finances, exercise, health, neighborhood, pets, home maintenance, free time, recreation outside the home) events. The possible range of frequency scores for social events is 0–10 and for non-social events, 0–43. Means and standard deviations for intensity ratings in this study were: social hassles (*M* = 1.37, *SD* = 0.04), social uplifts (*M* = 1.72, *SD* = 0.03), non-social hassles (*M* = 1.66, *SD* = 0.02), and non-social uplifts (*M* = 1.48, *SD* = 0.02). Means and standard deviations for frequency totals were: social hassles (M = 3.21, *SD* = 0.33), social uplifts (*M* = 4.26, *SD* = 0.47), non-social hassles (*M* = 15.36, *S D* = 1.45), and non-social uplifts (*M* = 12.40*,* *SD* = 1.21).

#### Global impression of change

The outcome assessment for overall change was measured with the Patient Global Impression of Change (PGIC) rating. The PGIC rating, assessed during the six-month follow-up phone interview of each participant, is based on seven levels of change ranging from very much worse to very much improved as it applied to the prior 6 months. Subjects who selected a PGIC rating of “very much worse,” “much worse,” or “somewhat worse” were assigned to the “worsened” subgroup. Individuals with an “unchanged” rating were assigned to the “unchanged” subgroup and participants who selected “very much improved,” “much improved,” or “somewhat improved” were assigned to the “improved” subgroup.

The PGIC rating, which provides a generalized view of the patient’s perception of overall change [26, 27], has shown construct validity in longitudinal studies of CFS on standard measures of fatigue and functioning [28]. In addition, patient-reported global outcomes of even modest improvement (the most commonly endorsed level in CFS), as opposed to no change or worsening, was associated with significantly improved fatigue (Fatigue Severity Scale) and functioning (SF-36 Physical Function Subscale) in a long-term outcome study [23]. The clinical relevance of the PGIC is assured as it allows patients to specify which constructs they judge as important for their health status [29]. Global assessment scales have been shown to be very sensitive to change, both positive and negative [30]. More generally, CFS prospective studies have often relied on the PGIC as a broad outcome measure of improvement or worsening [16].

### Power estimation

Sample size and power calculation issues were addressed in the primary study [13].

### Data analysis

Linear mixed effect models were utilized to examine and compare the linear trend of four qualitative types of hassles and uplifts over 26 weeks that included social intensity, non-social intensity, social frequency, and non-social frequency. With the assumption that hassles and uplifts exhibit linear trends over time, week was treated as a continuous variable. It was also hypothesized that patients in different outcome groups (improved vs unchanged vs worsened) would exhibit different weekly patterns; thus, an interaction term between week and outcome group was adjusted in our models. No other factors were adjusted in the regression models as this was an exploratory analysis.

Based on Akaike Information Criteria (AIC), the covariance structure to model correlations among longitudinal measurements from the same patient is selected from Compound Symmetry (CS), and first-order autoregressive (AR(1)), Toeplitz (TOEP), and Unstructured (UN). The coefficient of week, based on linear mixed effect models, was used to characterize the longitudinal pattern of behavioral measurement over 26 weeks. A coefficient > 0 suggests an increasing pattern and coefficient < 0 suggests a decreasing pattern. Sensitivity to missing data was assessed by analyzing the trends found using records from participants who had reported frequency data throughout the 26 weeks, since missing intensity data was due to the zero frequency.

Statistical analysis was performed using SAS 9.4 (SAS Institute Inc., Cary, NC) and significance level is set at 0.05.

## Results

The study sample consisted of 128 participants with global ratings at six-month follow-up of improved (29%; n = 37), unchanged (33%; n = 42) or worsened (38%; n = 49). No significant differences were found between groups for age, sex, or illness duration; however, work status was significantly reduced for the non-improved groups (*χ*^2^ = 819.72 (8); p < 0.001). Participant completion of weekly web diaries was excellent (95.6%).

**Fig. 1:**
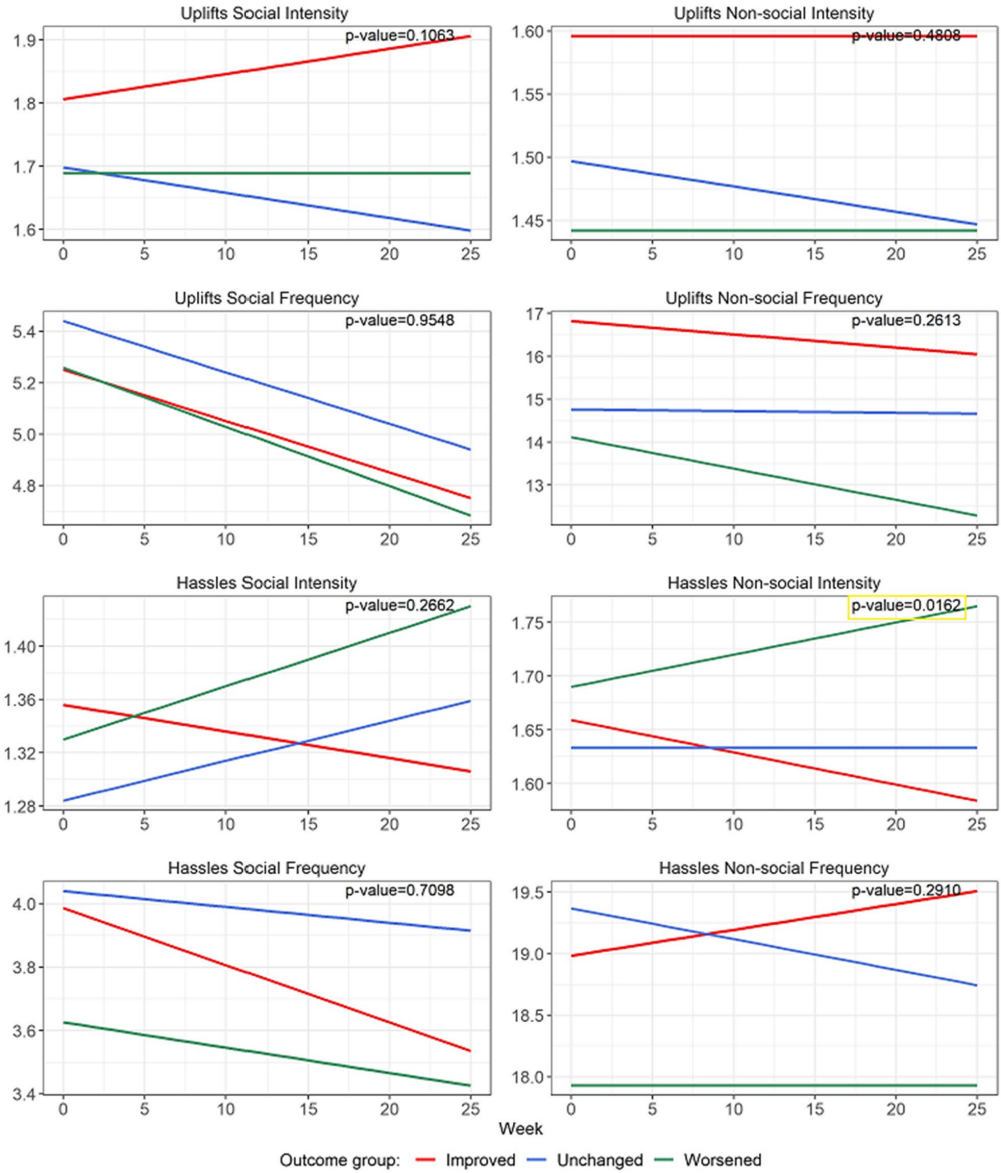
Linear regression lines of hassles and uplifts over 26 weeks based on linear mixed effect models. P-values indicate if there is a significant different *among* the three groups. This was found only for Hassles Non-social Intensity (right side, third down). The Uplifts Social Frequency graph (left side, second down) shows a significant downtrend in *all* three groups (p < 0.04)

Over 26 weeks, the longitudinal profile of *intensity of non-social hassles* (Fig. 1; Table 2) was significantly different across the 3 groups (p = 0.016). More specifically, the worsened group showed a significantly increasing pattern (weekly change = 0.003, p = 0.033) of non-social hassles intensity (Table 3), while the improved group evidenced a significant decreasing pattern (weekly change = − 0.003, p = 0.05).

**Table 2:** Type 3 *p*-values of explanatory variables in linear mixed models for each type of hassles and uplifts

Variable	Num DF	Den DF	F value	P-value^a^, ^b^	Covariance STRUCTURE
Uplifts social intensity					
Week	1	2791	0.00	0.9503	TOEP
Outcome group	2	125	0.75	0.4766	
Week * outcome group	2	2791	2.24	0.1063	
Uplifts non-social intensity					
Week	1	2852	0.38	0.5371	TOEP
Outcome group	2	125	2.25	0.1098	
Week * outcome group	2	2852	0.73	0.4808	
Uplifts social frequency					
Week	1	2881	17.44	** < 0.0001**	TOEP
Outcome group	2	125	0.10	0.9055	
Week * outcome group	2	2881	0.05	0.9548	
Uplifts non-social frequency					
Week	1	2881	4.02	**0.0450**	TOEP
Outcome group	2	125	1.84	0.1628	
Week * outcome group	2	2881	1.34	0.2613	
Hassles social intensity					
Week	1	2607	1.61	0.2052	TOEP
Outcome group	2	125	0.46	0.6296	
Week * outcome group	2	2607	1.32	0.2662	
Hassles non-social intensity					
Week	1	2856	0.02	0.8931	TOEP
Outcome group	2	125	0.31	0.7346	
Week * outcome group	2	2856	4.13	**0.0162**	
Hassles social frequency					
Week	1	2881	2.49	0.1145	TOEP
Outcome group	2	125	0.51	0.5996	
Week * outcome group	2	2881	0.34	0.7098	
Hassles non-social frequency					
Week	1	125	0.02	0.8968	UN
Outcome group	2	125	1.16	0.3165	
Week * outcome group	2	125	1.25	0.2910	

*TOEP* Toeplitz; *UN* Unstructured

^a^*p*-values were based on F-test from linear mixed models

^b^bolded *p*-values are statistically significant at *p* < .05

**Table 3:** Estimated coefficients of week based on linear mixed models for each type of hassles and uplifts (trend slopes)

Group	Estimated coefficient of week showing weekly change		P-value^a^, ^b^
	Coefficient	95% CI	
Uplifts social intensity			
Improved group	0.004	(− 0.002, 0.009)	0.1646
Unchanged group	− 0.004	(− 0.008, 0.001)	0.1057
Worsened group	0	(− 0.004, 0.004)	0.9480
Uplifts non-social intensity			
Improved group	0	(− 0.003, 0.004)	0.8602
Unchanged group	− 0.002	(− 0.005, 0.001)	0.1760
Worsened group	0	(− 0.003, 0.003)	0.9327
Uplifts social frequency			
Improved group	− 0.02	(− 0.039, − 0.001)	**0.0386**
Unchanged group	− 0.02	(− 0.037, − 0.003)	**0.0194**
Worsened group	− 0.023	(− 0.039, − 0.008)	**0.0033**
Uplifts non-social frequency			
Improved group	− 0.031	(− 0.098, 0.035)	0.3554
Unchanged group	− 0.004	(− 0.065, 0.057)	0.8866
Worsened group	− 0.073	(− 0.129, − 0.017)	**0.0110**
Hassles social intensity			
Improved group	− 0.002	(− 0.007, 0.004)	0.5620
Unchanged group	0.003	(− 0.002, 0.008)	0.2042
Worsened group	0.004	(− 0.000, 0.008)	0.0794
Hassles non-social intensity			
Improved group	− 0.003	(− 0.007, 0.000)	0.0510
Unchanged group	0	(− 0.003, 0.003)	0.9860
Worsened group	0.003	(0.000, 0.006)	**0.0330**
Hassles social frequency			
Improved group	− 0.018	(− 0.042, 0.006)	0.1444
Unchanged group	− 0.005	(− 0.027, 0.017)	0.6814
Worsened group	− 0.008	(− 0.029, 0.012)	0.4210
Hassles non-social frequency			
Improved group	0.021	(− 0.023, 0.066)	0.3500
Unchanged group	− 0.025	(− 0.063, 0.013)	0.1937
Worsened group	0	(− 0.035, 0.034)	0.9817

^a^*p*-values were based on T-test from linear mixed models

^b^bolded *p*-values are statistically significant at *p* < .05

The trend slope (Fig. 1; Table 3) for *frequency of social uplifts* significantly decreased over time in all three groups as follows: improved (weekly change = − 0.02, p = 0.039), unchanged (weekly change = − 0.02, p = 0.019), and worsened (weekly change = − 0.02, p = 0.003). By comparison, only the worsened group showed a significantly decreasing pattern in the frequency of *non*-social uplifts (weekly change = − 0.07, p = 0.011).

Comparing the trend slope across groups (Fig. 1; Table 3), the improved group and worsened group presented significantly different patterns of change for non-social hassles intensity (improved vs worsened groups: difference of coefficient of week = − 0.006, p = 0.004). No other significant trends were found for uplifts or hassles.

For missing data, the sensitivity analyses on *intensity* included 94 (73.44%) participants for social uplift intensity, 109 (85.16%) for nonsocial uplift intensity, 68 (53.12%) for social hassle intensity, and 112 (87.5%) for nonsocial hassle intensity. These participants had reported all hassles or uplifts over the 26 weeks without missing data on intensity. The linear trend of *intensity of non-social hassles* was significantly different across 3 groups (p = 0.0051). It was significantly decreased in the improved group (week change = − 0.004, p = 0.018) but significantly increased in worsened group (week change = 0.003, p = 0.0248). There existed a significant difference of trend slope of intensity of non-social hassles between improved and worsened groups (improved vs worsened: difference of week change = − 0.008, p = 0.001). Other types of intensity were not significantly changed over time and the overall results were not altered (see Table 4).

**Table 4:** Estimated difference of the coefficient of week based linear mixed models for each type of hassles and uplifts (difference of trend slope)

Group	Estimated difference in the coefficient of week		P-value^a^, ^b^
	Coefficient difference	95% CI	
Uplifts social intensity			
Improved vs unchanged	0.007	(0.001, 0.014)	**0.0346**
Improved vs worsened	0.004	(− 0.003, 0.011)	0.2636
Unchanged vs worsened	− 0.004	(− 0.010, 0.003)	0.2519
Uplifts non-social intensity			
Improved vs unchanged	0.002	(− 0.002, 0.007)	0.2989
Improved vs worsened	0	(− 0.004, 0.005)	0.9353
Unchanged vs worsened	− 0.002	(− 0.006, 0.002)	0.2924
Uplifts social frequency			
Improved vs unchanged	0	(− 0.026, 0.026)	0.9948
Improved vs worsened	0.003	(− 0.022, 0.028)	0.8013
Unchanged vs worsened	0.003	(− 0.020, 0.026)	0.7933
Uplifts non-social frequency			
Improved vs unchanged	− 0.027	(− 0.117, 0.063)	0.5589
Improved vs worsened	0.042	(− 0.045, 0.129)	0.3488
Unchanged vs worsened	0.069	(− 0.014, 0.152)	0.1056
Hassles social intensity			
Improved vs unchanged	− 0.005	(− 0.012, 0.003)	0.2009
Improved vs worsened	− 0.006	(− 0.013, 0.001)	0.1190
Unchanged vs worsened	− 0.001	(− 0.007, 0.006)	0.8064
Hassles non-social intensity			
Improved vs unchanged	− 0.003	(− 0.008, 0.001)	0.1495
Improved vs worsened	− 0.006	(− 0.010, − 0.002)	**0.0042**
Unchanged vs worsened	− 0.003	(− 0.007, 0.001)	0.1457
Hassles social frequency			
Improved vs unchanged	− 0.014	(− 0.046, 0.019)	0.4191
Improved vs worsened	− 0.01	(− 0.041, 0.022)	0.5423
Unchanged vs worsened	0.004	(− 0.026, 0.034)	0.8087
Hassles non-social frequency			
Improved vs unchanged	0.046	(− 0.012, 0.105)	0.1207
Improved vs worsened	0.022	(− 0.035, 0.078)	0.4511
Unchanged vs worsened	− 0.025	(− 0.076, 0.027)	0.3420

^a^p-values were based on T-test from linear mixed models

^b^bolded *p*-values are statistically significant at *p* < .05

## Discussion

In this six-month observational study of individuals with CFS involving 26 weekly assessments, only a few clear differences were found between self-report worsened as compared to improved subjects on the dimensions of behavioral uplifts and hassles. The worsened group showing an increasing pattern for non-social hassles, while the improved group evidenced a decreasing pattern. In addition, the frequency of social uplifts significantly decreased in all three group across the six months assessment interval. However, only the worsened group showed a significant decrease in *non-*social uplifts frequency.

### Uplifts deficits and worsening

Our finding of a downtrend in the frequency of specifically “non-social” uplifts in the worsened group may have some overlap with our earlier study [13] in which a lower intensity of uplifts predicted self-report non-improvement (unchanged and worsened) in individuals with CFS. Perhaps non-social activities are more salutary as they are more readily available, more manageable, and potentially less energy-depleting than socially positive interactions [30]. In general, excessive fatigue is triggered in CFS in response to even minor activities [31], regardless of valence, and thus it may be challenging for patients to thread the needle to eventual illness improvement via greater uplifts, fewer hassles, and other positive self-management activities. Even if successful, relatively small improvements in illness symptoms may result, as suggested by the modest 15% downward trend of weekly fatigue ratings in the CFS improver group recorded over six months in the primary study [13]. Not surprisingly, in the current observational study, a far lower percentage of individuals rated themselves as improved as compared to two previous behavioral self-management trials in CFS [24, 32].

Speculatively, these findings could reflect an ongoing change process, only partially captured in this six-month study that may inform specific behavioral pathways to worsening and improvement. Fewer pleasant experiences in CFS have been associated with higher fatigue and lower functioning over 18 months [3]. In addition, our data revealed that the intensity of non-social hassles increased in worsened subjects and decreased in improved subjects (Fig. 1). More broadly, negative social events have been associated with higher daily fatigue in chronically fatiguing illnesses, e.g., fibromyalgia, rheumatic arthritis [8], suggesting that social interactions may play a role in determining the magnitude of ongoing fatigue experienced by those with chronic fatigue and pain [9].

As compared to the primary study [13], our expanded range of significant findings regarding uplifts and hassles as possible outcome predictors may be explained in part by several design changes in the present study: (1) the unit of analysis was weekly scores on the CHUS, rather than single 26-week means used for each subject in the primary study; (2) the use of separate categories for unchanged and worsened outcomes rather than the more generic non-improvement construct; and (3) the subdivision of hassles and uplifts into social and social sub-categories. Overall, the current analysis represents a fine-grained examination of hassles and uplifts in the experiences of individuals with CFS, which was likely to identify more precise and potentially more informative outcome predictors.

### Clinical implications

Although the salient illness variable of fatigue was not assessed as an outcome variable in this study, a critical element of improved outcomes in CFS is based on the patients’ personal efforts to effectively manage their illnesses such that well-being and functioning are maximized. In the absence of curative treatments, this is perhaps the most beneficial type of outcome that can be realistically achieved. In addition, perceived global improvement in CFS, even if modest, has been associated with significantly reduced fatigue and higher functioning over a two-year observational period [23]. Perhaps a clinical focus on selectively assigning uplifts and limiting hassles, as suggested by our findings, could be utilized as a straightforward approach to facilitating improvement in CFS. Potential therapeutic changes in target areas, as suggested by our hassles and uplifts findings, do not necessarily have to be of high magnitude to result in overall improvement.

Although not an intervention or controlled trial, clinically relevant findings in this observational study suggest the potential importance of uplifts to perceived global improvement in this difficult-to-treat illness. Uplifts can be a focus of behavioral management [12] if the clinician collaboratively identifies with the patient pleasant, enjoyable, low-effort activities that are often lacking in the lives of individuals with debilitating CFS [33, 34]. This may have relevance to CFS pathophysiology given that a large biobehavioral study in healthy adults suggested that the absence of positivity in daily life may be particularly consequential for inflammation [6].

Examples of positive events that could be applied clinically in CFS include listening to an inspirational speaker, going to a concert, watching ducks on a pond, sharing a special moment with a spouse or friend, or any other moderately pleasant activity that does not trigger long-duration symptom worsening. To generate ideas, the patient can be asked to make a list of 10 pleasant low-effort activities. Once these possibilities are identified, a flexible schedule is developed so that the patients can participate in pleasant activities at least several times a week. Although illness-related restrictions may have reduced opportunities to engage in pleasant experiences [12], about 1/3 of our (often homebound) study participants were apparently able to engage in uplifting activities and reduce the intensity of their hassles over several months to the point where they rated themselves as “improved”.

Our findings regarding worsening illness in association with fewer non-social uplifts are also consistent with developing beneficial treatment targets in two evidenced-based therapies, Behavioral Activation and Acceptance and Commitment Therapy. Although not described as “uplifts”, both treatments consist of helping patients to identify and clarify chosen values [35, 36], such as who is important to them (e.g., friends, family), what is important to them (e.g., physical and mental health, companionship) and what qualities of action they want to embody (e.g., loyalty, trustworthy, kindness). Once values are identified, clinicians help patients start to behave in ways in line with the chosen values in pursuing the most rewarding or intense socially uplifting activities by identifying *who* (i.e., spending time with grandchildren) or *what* (non-social) is in that category rather than participating in social gatherings that do not have the same valence (i.e., spending time with an acquaintance).

Furthermore, by encouraging patients to focus on chosen values, it is possible that they may also be less likely to be bothered by hassles, which were significantly greater in our worsened subjects. Both Behavioral Activation and Acceptance and Commitment Therapy have also been found to be associated with improved well-being [37, 38]. This may explain why in the current study, improved subjects experienced significantly less intense non-social hassles than their worsened counterparts. Future work should continue to examine the effect of improving the frequency of uplifts, or values, in patients with CFS on physical and emotional well-being.

### Limitations

As this study was not a randomized treatment trial, clinical approaches to illness improvement may be suggested but not definitively recommended. This is due to reliance on observational and correlational data, limiting causal conclusions and generalizability. In addition, recruitment bias is likely as participants were obtained largely from CFS support group websites whose readership under-represents non-white individuals. Participant demographics heavily favored white females (with long-term illness), although the age range (largely 40s) is consistent with prevalence studies (e.g., [39]). That said, prospective subjects with a potentially much wider range of illness severity and disability were eligible for the study as no travel or costs were involved.

Furthermore, weekly trajectories of uplifts and hassles were grouped and analyzed by global change categories which may have obscured more nuanced individual patterns and the influence of mental health comorbidities. Comorbid mental health conditions, e.g., depression and anxiety, could be indirectly examined by assessment of daily positive and negative affect with techniques such as ecological momentary assessment [40] in order to determine their influence on the frequency and intensity of uplifts and hassles. In addition, underlying mechanisms could be explored. A recent empirical study [41] of genetic susceptibility to daily events in young female participants found that a serotonin gene variant, the serotonin-transporter-linked polymorphic region (*5-HTTLPR)* played a role in general reactivity to both positive and negative events. Perhaps this gene variant contributed to the reported experience of uplifts and hassles in CFS participants that in turn influenced worsened or improved outcomes.

Apart from mechanisms, the potential interaction of uplifts and hassles merits attention. A recent study of uplifts and hassles in relation to unhealthy eating habits [42] found that daily uplifts buffered the effects of daily hassles on between-meal snacking resulting in fewer episodes of unhealthy snacking. These findings suggest that the negative effects of daily hassles on worsened short-term outcomes can potentially be mitigated by the experience of daily uplifts. Finally, the use of weekly self-report measures and web diaries may be subject to bias. The end-of-week recall covering the previous 7 days as compared to daily assessments tends to show more intense symptoms and lower quality of life, although Pearson’s correlations are strong [43, 44]. However, the use of daily assessments, although potentially more accurate, increases participant burden and as such were not used in this study.

## Conclusions

Given the controversies regarding the safety and efficacy of well-publicized graded activity interventions in CFS [45], our alternate or perhaps complementary focus on behavioral uplifts and hassles as possible improvement predictors may be clinically useful. For instance, one path to behavioral improvement in CFS that is supported by our findings may be through the scheduling of more frequent non-social uplifts, and perhaps reducing the emotional impact of intense hassles (cf., [11]). These commonly experienced minor events can be voluntarily modified and therapeutically managed, with less potential adverse consequence than standard behavioral approaches in the service of improving well-being and outcomes in CFS.

Future research might also explore underlying mechanisms linking uplifts and hassles to illness worsening and identify potential intervention targets with consideration of comorbid mental health conditions. Focusing on uplifts and hassles in behaviorally oriented treatments may be a promising strategy to improve outcomes in individuals with CFS. To strengthen the evidence base and generalizability, randomized treatment trials in more demographically diverse populations would be beneficial. The study's findings may inform the development of safer, tailored interventions for CFS that could also explore synergies with other evidence-based therapies and ultimately pave the way for future clinical research and improved patient care.

## Data Availability

The data that support the findings of this study are available from the corresponding author (FF), upon reasonable request.
